# Trajectories of pain and cognitive function: 22 years of evidence in mid-to-later life

**DOI:** 10.1097/j.pain.0000000000004052

**Published:** 2026-06-24

**Authors:** Salomé Andres, Simon R. Cox, Chloe Fawns-Ritchie

**Affiliations:** a Advanced Care Research Centre Academy, College of Science and Engineering, University of Edinburgh, United Kingdom; b Lothian Birth Cohorts, Edinburgh Futures Institute, University of Edinburgh, Edinburgh, United Kingdom; c Lothian Birth Cohorts, Department of Psychology, University of Edinburgh, Edinburgh, United Kingdom; d Department of Psychology, The University of Edinburgh, Edinburgh, United Kingdom

**Keywords:** Pain severity, Cognitive decline, Longitudinal study, Older adults, Latent growth curve

## Abstract

Chronic pain has been identified as a risk factor for cognitive decline in later life. However, most studies measure pain at a single time point, and none have investigated whether variations in pain severity are associated with changes in cognitive function over time. This project aimed to assess the relationship between individual-level change in pain severity and decline in cognitive function over time. We used data from the English Longitudinal Study of Ageing, a cohort of nationally representative middle aged and older adults. Pain severity was measured at each wave using a 4-point scale (none, mild, moderate, and severe), and cognitive function was assessed using 3 objective tests. We applied latent growth curve modelling, a method for longitudinal analysis, to 19,376 study participants' data collected over 11 waves, spanning more than 20 years, to examine the relationship between initial level and change of both pain and cognitive function. Adjusting for age and sex, worsening chronic pain severity was associated with accelerated decline in a general measure of cognitive function (β = −0.053, *P* = 0.039). However, when additionally adjusting for ethnicity, socioeconomic status, and comorbid chronic conditions, this association was attenuated to nonsignificance (β = −0.025, *P* = 0.365). Greater initial pain severity was associated with steeper decline in cognitive function even in the fully adjusted model (β = −0.104, *P* < 0.001). Our study suggests that baseline level of pain severity but not worsening pain severity is associated with steeper decline in cognitive function over time.

## Abstract 2

Supplemental Digital Content is Available in the Text.

Age- and sex-adjusted analyses find that higher baseline and worsening pain severity predict faster cognitive decline; only baseline pain remains significant after full adjustment.

## 1. Introduction

Chronic pain and cognitive decline represent 2 major public health challenges, particularly in ageing populations. Chronic pain affects an estimated 35% to 51% of adults in the United Kingdom, rising to over 60% in adults aged over 75 years.^14^ Concurrently, key cognitive functions such as memory and processing speed decline with increasing age,^51,53^ although rates of decline vary between individuals.^11,19,55,63^ Identifying potential risk factors of accelerated cognitive decline is crucial, and chronic pain's contribution has not been wholly explored.

Individuals with chronic pain tend to perform worse on various cognitive tests, including memory, executive function, and processing speed, compared with those without pain.^7,8,21,31,34,65^ This cross-sectional evidence cannot distinguish between stable between-person differences and within-person changes in cognitive function over time, nor establish the temporal ordering of pain and cognition. Longitudinal findings are mixed, with some studies reporting that presence of pain, greater pain severity, or more years of pain duration are associated with steeper cognitive decline or incidence of cognitive impairment.^5,48,60^ However, a recent meta-analysis^1^ of 10 longitudinal studies (57,495 older adults) reported that persistent pain at baseline was not associated with incident cognitive decline up to 11.8 years later (RR = 1.05; 95% CI 0.92-1.21).

Although these longitudinal studies measure cognitive function longitudinally, most evaluate pain only once, despite evidence of the temporal instability of pain presence and severity.^12,13,16,29^ Cross-sectional designs are particularly vulnerable to measurement error and confounding (reverse causation, time-invariant confounding, dynamic exposure misclassification), particularly in the context of a subjective measure such as pain, which is standardised internally by participants. Longitudinal studies, by capturing these processes dynamically, minimise these confounds and improve reliability, inferential precision, and power.^43,44^ As pain may emerge, intensify, or remit during follow-up, modelling longitudinal change in *both* pain and cognitive function over time offers a more stringent test of a causal hypothesis with correlational data. Milani et al.^33^ analysed 2 waves of data over 4 years (n = 2349) using cross-lagged models and found that higher-than-expected pain interference (ie, whether pain interfered with daily activity) at follow-up was associated with lower-than-expected scores on a brief cognitive screening test, after controlling for baseline scores (β = −0.07, *P* < 0.01). Another study^20^ approached this question using group-based trajectory modelling, which categorised participants in 3 distinct groups (low-stable, high-stable, and moderate-increasing) based on their changes in the probability of presence or absence of pain across time rather than fluctuations in pain severity. Using linear mixed-effect models, the authors found that the moderate-increasing group exhibited steep decline on executive function (β = −0.005 SD/year, *P* = 0.012) and global cognition (β = −0.014 SD/year, *P* < 0.001); however, this method assumes all group members share the same trajectory, neglecting within-person variation.

The current study addresses existing gaps by applying latent growth curve modelling (LGCM) and leveraging 22 years of data collected on both pain severity and cognitive function from 19,376 community-dwelling adults. This approach allows for the investigation of the effect of *within-person* changes in pain severity over time on concurrent *within-person* changes in cognitive function.

## 2. Methods

### 2.1. Study population

This study leverages data from the English Longitudinal Study of Ageing (ELSA),^3,54^ a population-based cohort study designed to be representative of community-dwelling adults aged 50 years and older residing in England. Data collection commenced in 2002 (wave 1 n = 12,099), and participants were recruited from responders to the Health Survey for England^36^ over the age of 50 and their spouse. Participants have been followed up approximately every 2 years, and the sample has been refreshed at waves 3, 4, 6, 7, 9, 10, and 11 to ensure representativeness is maintained. To date, 11 waves of data have been collected from a total of 23,259 participants. Because the present study aims to investigate cognitive decline in mid-to-later life, we used data from the 19,376 participants who were over the age of 40 in 2002, which marks the start of data collection for wave 1.

Data were collected via face-to-face interviews conducted in the participant's own homes and via self-completion questionnaires. Information collected included sociodemographic, health, and lifestyle reports. Although all participants were recruited whilst they resided in the community, a small portion of subsequent interviews took place in institutions (0.6% in our sample) if the participant moved to a care home or similar establishment. Detailed descriptions of the study design and data collected are reported elsewhere.^64^

Data were downloaded from the UK Data Service in May 2025. English Longitudinal Study of Ageing has received ethical approval for all waves of data collection (most recently from South Central–Berkshire Research Ethics Committee on 22nd May 2023; REF: 23/SC/0112).

### 2.2. Measures

#### 2.2.1. Pain severity

At each wave, participants were asked “Are you often troubled with pain?” Participants who answered “yes” were then asked “How bad is the pain most of the time? Is it mild, moderate, or, severe?”. To capture pain severity in the current study, we created a four-point ordinal scale (none, mild, moderate, severe) from these answers.

#### 2.2.2. Cognitive function

The current study used scores on 3 of the cognitive tests. Memory was assessed with a recall test (word-list learning): Participants heard a list of 10 words that they had to recall immediately (immediate recall) and after a short delay (delayed recall), during which they completed other cognitive tests. Scores on the immediate and delayed recall tests were strongly correlated (*r* = 0.80, *P* < 0.05) and combined into a word recall variable by summing these results (range 0-20). Data for the recall test were available at all 11 waves. Executive function was assessed with animal naming (word-finding), a category fluency test where participants were asked to name as many animals as possible in 60 seconds. The score was the number of unique animals named within the time limit. Animal naming was collected at all waves, except wave 6. Finally, processing speed was assessed with letter cancellation, which involved participants crossing out as many Ps and Ws on a sheet of paper as fast as possible in 60 seconds. The score was the number of letters correctly scored out within the time limit. Letter cancellation was only assessed at waves 1 to 5.

Scores of 0 in animal naming (n = 727) and letter cancellation (n = 28) were set to missing as these likely indicate the participant did not complete or understand the task. After visual inspection of the distribution of all 3 cognitive tests (supplemental digital content, http://links.lww.com/PAIN/C534), scores 4 standard deviations above the mean for animal naming (4 SD = 54; n = 21) were set to missing as these results are extremely high given the time limit, and potentially suggest an administration or data entry error. There were no outliers in the recall tests plots, and the letter cancellation appeared to have a heavy-tailed distribution rather than isolated anomalies, so no further alterations were made to either variable.

#### 2.2.3. Covariates

Age, sex, ethnicity, socioeconomic status, and comorbidities were used as covariates. Unless otherwise specified, covariate data were taken from the first wave in which participants provided data. Age was measured in years, and participants under 40 in 2002 (at wave 1) were removed from the dataset (n = 3883). To avoid disclosure due to small numbers, ELSA set the age of all participants over 90 years to a value 99. Here, we recoded these individuals' ages to 91 years. To improve precision of the age variable, we used information from ELSA wave 0 (ie, the Health Survey for England 1998-2001) for participants whose age was recorded at 99 in wave 1 (n = 62) and added 4 years (time elapsed between waves 0 and 1) to their reported age at wave 0. Finally, the age variable was standardised (mean = 0; SD = 1) before being added to the model to help convergence. Sex was recorded as a binary, male, or female. The ethnicity variable was collapsed by ELSA to “white” and “non-white” to avoid disclosure. Socioeconomic status was based on the 8-point scale of the National Statistics Socio-Economic Classification ranging from “higher managerial and professional occupations” to “never worked or long-term unemployed”^38^ and determined for each participant individually. Comorbidity variables were derived from responses to the question “has a doctor ever told you that you have (or have had) [list of medical conditions]”. These include diabetes (or high blood sugar), hypertension (high blood pressure), heart disease (angina, a heart attack, congestive heart failure, an abnormal heart rhythm or any other heart trouble), lung disease, stroke, and cancer or a malignant tumour (excluding minor skin cancers). Participants were classified as having a specific health condition if they ever reported it, at any wave of data collection.

### 2.3. Statistical analysis

To estimate longitudinal trajectories of pain and cognitive function, we used LGCM, a structural equation modelling (SEM) technique, which estimates both individual- and group-level trajectories over time. Conceptually, SEM allows researchers to investigate variables, which are unobservable and measured indirectly by multiple indicators. Latent growth curve modelling explicitly parses the longitudinal data into an initial starting point (a latent intercept) and change over time (a latent slope).^27^ This specific framework allows for the relationship between intercepts and slopes of both pain severity and cognitive decline to be estimated and their associations tested within the same simultaneous model.

Using observed pain severity scores across 11 waves, we derived 2 latent variables, which capture the intercept (essentially pain severity level at baseline) and slope (linear rate of change in pain severity across the 11 waves) for each participant. To translate the 4 observed ordinal values to a latent variable with maximum likelihood estimator, the model determined 3 thresholds, which are the cut-off points on the underlying latent variable that mark where the probability of endorsing one ordinal category shifts to the next.

**Figure 1.**
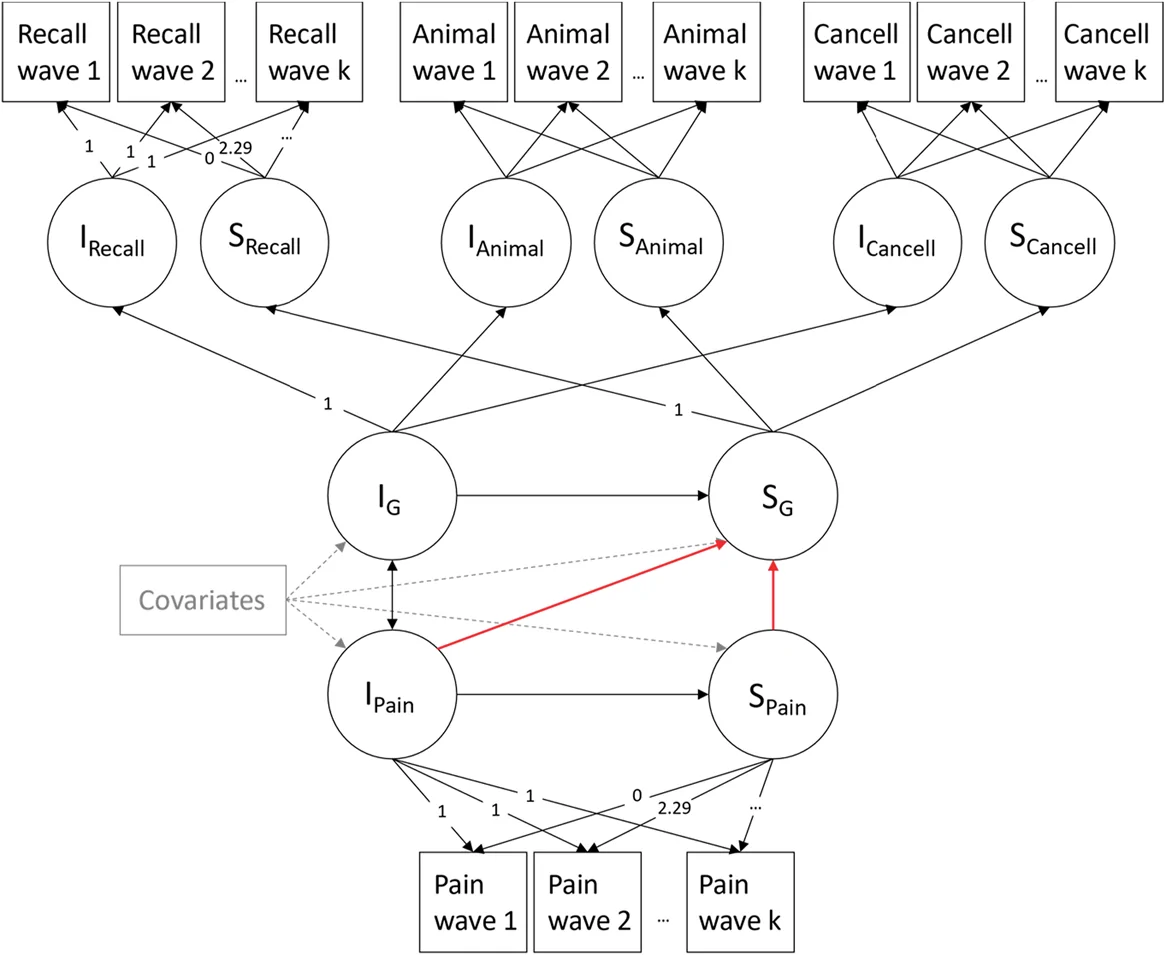
Simplified path diagram of the parallel process latent growth curve model of pain severity and general cognitive function. Circles are latent factors, squares represent observed variables, single-headed arrows are regression paths, and double-headed arrows are correlation paths. The red arrows are the focus of our hypothesis. Dashed grey lines are regression paths for covariates. I = Intercept, S = Slope, G = general cognitive function, Animal = Animal naming test, Cancell = Letter cancellation test, Recall = Word recall test. For illustrative purposes, the loadings for animal naming and letter cancellation are not displayed; however, these are the same as the loading for recall and pain. After the marker variable approach, the word recall test's loadings on general cognitive function intercept and slopes were set to 1, and the means of intercept and slope of the word recall test were set to 0.

To model cognitive function, we used the hierarchical “factor-of-curves”^32,62^ method: for each individual cognitive test (word recall, animal naming, and letter cancellation), we modelled a latent intercept (cognitive level at baseline) and slope (rate of change in the cognitive test across the 11 waves) using the observed cognitive test scores across waves. We imposed a superordinate latent measure of general cognitive function (“*g*”; top half of Fig. 1) to reflect the well-known correlational structure across cognitive tests and their correlated changes over time.^10,57^ To allow identification, the marker variable approach was used, where the loading of the word recall latent intercept and slope on higher-order factors was fixed at 1 and the mean intercept and slope of recall set to 0. The slope loadings for each cognitive test reflected the mean time in years since wave 1 (0, 2.29, 4.29, 6.25, 8.29, 10.21, 12.21, 14.21, 16.29, 19.79, and 21.59, estimated from the month and year of data collection at each wave), fixing the slope estimated scale to change in standard deviation units per year.

Model fit was assessed using root-mean-square error of approximation (RMSEA) and comparative fit index (CFI), with RMSEA values of ≤0.08 and CFI values ≥0.90 considered as indicating acceptable fit.^27^ Details on the factor-of-curves building process can be found in supplemental digital content II (http://links.lww.com/PAIN/C534).

To estimate the relationship between initial level (intercept) and change over time (slope) of pain and cognitive function, we fitted 3 parallel process LGCMs. All 3 models included a correlation between the intercept of pain and the intercept of cognitive function; a regression path from intercept of pain to the slope of pain; and regression paths from the intercept of cognitive function, the intercept of pain, and the slope of pain to the slope of cognitive function. The structure of the regression paths is based on our hypothesis and temporality of the effects. We did not want to assume a directional relationship between baseline pain severity and baseline cognitive function because both directions have been reported and correlational associations have previously been suggested in the literature.^15,28^ Model 1 was unadjusted, model 2 adjusted for age and sex, and model 3 additionally adjusted for socioeconomic status, ethnicity, and comorbidities. Covariates were all considered time invariant and added using regression paths from each covariate to the intercepts and slopes of both pain and cognitive function. A simplified path diagram of the parallel process model is shown in Figure 1. Here, we are interested in how pain relates to cognitive decline. Our primary focus is the regression path from the slope of pain to slope of cognitive function, and the secondary focus is the regression path from intercept of pain to slope of cognitive function (both paths shown in red in Fig. 1).

Because we modelled these regression paths simultaneously, the resultant path estimates for the intercept and the slope of pain on cognitive declines are mutually conditioned. That is, the contributions of pain at baseline on cognitive change is independent of the statistical contribution of changes in pain, and vice versa. Thus, where both paths are significant, this indicates unique, independent contributions of both how much pain one has to begin with, and how much that changes over time, to cognitive ageing trajectories. We also ran separate supplementary models where the intercept and slope of pain are not mutually conditioned on each other to highlight the additive process of our model design.

Missing data is common in longitudinal studies, often resulting from participants not answering questions, intermittently skipping waves, or dropping out of the study completely. The English Longitudinal Study of Ageing refreshes its sample by adding new participants at certain waves to maintain its representativeness of the English population aged over 50 years. Much of the missing data in our analysis arose by design: refresher participants had missing data on the previous waves, the animal naming test was not administered at wave 6, and the letter cancelling test was only assessed up to wave 5. Patterns of missingness and rate of attrition for the pain variable are reported in supplemental digital content III (http://links.lww.com/PAIN/C534). In this study, we used the maximum likelihood estimator with robust standard errors (MLR), specifying that the pain severity variables were ordered categorical. The *probit* link function was used, which is appropriate for categorical data with more than 2 levels. With this estimator, our model ran under Full Information Maximum Likelihood (FIML), which uses all available data points to estimate model parameters. FIML assumes our data are missing at random (MAR)^50,52^; that is, after accounting for the observed variables in the model, the probability that a value is missing does not depend on the unobserved values. This assumption allows FIML to produce unbiased parameter estimates by using all available data points. If there were missing data on the covariates, the participants would automatically be removed from the study by the maximum likelihood estimator using listwise deletion. This led to a small discrepancy in sample size in our 3 models (model 1 n = 18,880, model 2 n = 18,861, model 3 n = 18,462).

To achieve convergence of the categorical LGCM of pain severity under the MLR estimator and *probit* link function and due to low representation in the *severe* category of pain severity (cross-wave average n = 685 [8%]), we applied partial invariance of the thresholds: the third threshold was free to vary over time whilst the first and second thresholds were held invariant. It is not possible to compute conventional fit indices such as RMSEA and CFI after including the latent factors derived from the categorical pain variable. However, inspection of the cognitive test loadings on the higher-order factors confirmed they remained similar to the factor-of-curves LGCM of general cognitive function before its integration into the parallel process model (see supplemental digital content IV for detailed table, http://links.lww.com/PAIN/C534).

We also investigated the associations between the intercepts and slopes of pain and each individual cognitive test, following the same pattern of correlations and regression paths as the primary analysis but without the higher-order general cognitive function factors (illustrative path diagram shown in supplemental digital content V, http://links.lww.com/PAIN/C534).

We used R version 4.5.1^42^ in the RStudio environment version 2025.09.1+401^41^ for data preparation and plotting. MPlus version 8.11^35^ was used for SEM analysis. The R package MPlusAutomation version 1.2^17^ allowed us to transfer factor scores from MPlus to R. See Acknowledgements for code repository details.

## 3. Results

### 3.1. Descriptive statistics

**Figure 2.**
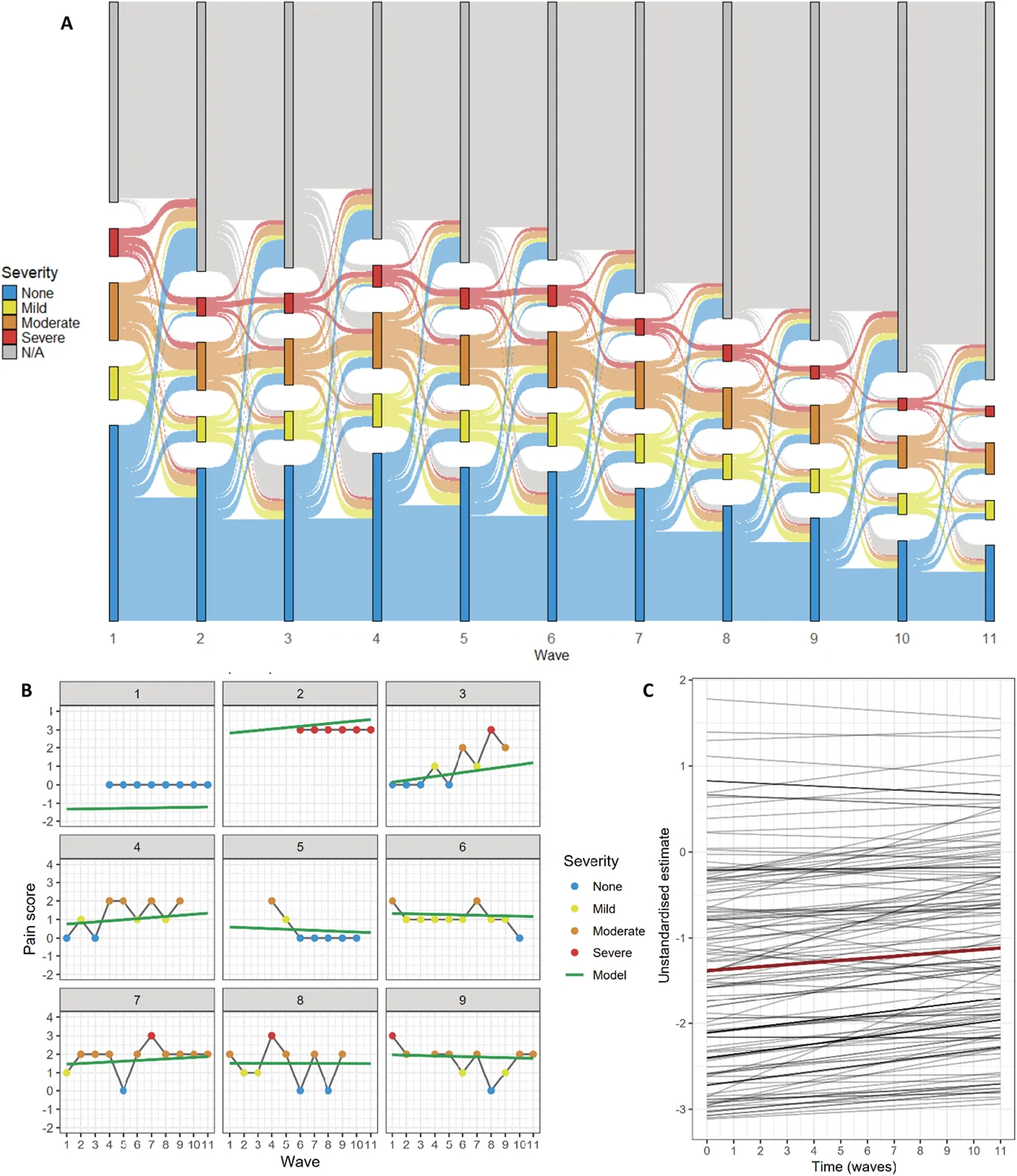
Trajectories of pain severity. (A) Sankey diagram of pain severity showing category change over time across 11 waves. (B) Nine participants' pain reports over 11 waves and their model-estimated trajectories. These 9 participants were selected for illustrative purposes and are not systematically representative of the study sample. The green lines labelled “Model” are the individual trajectories (intercept and slope) estimated by our model for each participant. (C) The estimated trajectory (intercept and slope) of pain in a random sample of 150 participants estimated by the measurement model (shown in grey), and the model's mean estimate shown in dark red (intercept β = −1.381, slope β = 0.024).

Detailed descriptive results of the pain and cognitive function variables at each wave are shown in Table 1 (plots showing their distribution are in Fig. 2A and supplemental digital content I, http://links.lww.com/PAIN/C534). The combined sample of 19,376 participants had a mean age of 58.84 years at wave 1 and 53% were female. Other participant characteristics are reported in Table 2, and more details on wave-by-wave characteristics are available in supplemental digital content VI, http://links.lww.com/PAIN/C534.

**Table 1:** Descriptive statistics on the 3 cognitive tests and reports of pain severity.

Wave number	1	2	3	4	5	6	7	8	9	10	11
Time since wave 1*	0	2.29	4.29	6.25	8.29	10.21	12.21	14.21	16.29	19.79	21.59
N	12,039	9380	9662	10,928	10,153	10,372	9195	8060	7286	6010	5690
Recall											
N	11,645	9187	9359	10,416	9562	9680	8432	7425	6635	5307	5018
Mean	9.46	9.94	10.29	10.39	10.41	10.65	10.46	10.57	10.47	10.40	10.62
SD	3.59	3.62	3.70	3.65	3.73	3.72	3.79	3.79	3.71	3.54	3.53
Animal naming											
N	11,618	9150	9312	10,366	9461		8322	7382	6598	5339	5071
Mean	19.38	19.90	20.31	20.78	20.94		21.29	21.59	22.01	22.18	22.37
SD	6.35	6.47	6.78	6.76	6.69		6.87	6.99	7.17	7.29	7.28
Letter cancellation											
N	11,237	8913	8897	9341	8935						
Mean	18.74	18.44	18.94	19.10	18.89						
SD	5.96	5.85	5.74	5.47	5.43						
Pain severity											
None											
N	7343	5749	5843	6315	5766	5608	4986	4349	3860	3034	2850
%	62.0	62.1	62.1	60.4	60.1	57.9	59.0	58.1	57.8	55.5	55.0
Mild											
N	1252	972	1085	1242	1168	1227	1069	949	876	786	718
%	10.6	10.5	11.5	11.9	12.2	12.7	12.6	12.7	13.1	14.4	13.9
Moderate											
N	2177	1817	1725	2090	1873	2084	1771	1545	1451	1204	1202
%	18.4	19.6	18.3	20.0	19.5	21.5	20.9	20.7	21.7	22.0	23.2
Severe											
N	1077	714	754	816	792	770	628	637	493	445	411
%	9.1	7.7	8.0	7.8	8.3	7.9	7.4	8.5	7.4	8.1	7.9

* Time since wave 1 is measured in years and corresponds to the first order factor loadings.

**Table 2:** Covariates descriptive characteristics.

	Mean (SD)/N (%)	Missing N (%)
Age	58.84 (11.9)	7 (<0.1%)
Sex		20 (0.1%)
Female	10,355 (53.4%)	
Male	9021 (46.6%)	
Ethnicity		114 (0.6%)
White	18,420 (95.1%)	
Non-White	956 (5.9%)	
Socioeconomic status		672 (3.5%)
SES 1	1933 (10.0%)	
SES 2	3892 (20.1%)	
SES 3	2893 (14.9%)	
SES 4	1943 (10.0%)	
SES 5	1907 (9.8%)	
SES 6	3158 (16.3%)	
SES 7	2746 (14.2%)	
SES 8	232 (1.2%)	
Comorbidities		
Diabetes	1121 (5.8%)	13 (<0.1%)
Hypertension	3444 (17.8%)	13 (<0.1%)
Heart disease	2402 (12.4%)	15 (<0.1%)
Lung disease	839 (4.3%)	14 (<0.1%)
Stroke	712 (3.7%)	13 (<0.1%)
Cancer	1285 (6.6%)	13 (<0.1%)

Comorbidities are considered if participant ever reported being told by a clinician that they have the diagnosis at any wave. Values are mean (SD) for age and N (%) for all other variables.

SES, Socioeconomic Status based on the National Statistics Socio-Economic Classification.^38^

### 3.2. Measurement models

#### 3.2.1. Pain severity univariate latent growth curve modelling

There was substantial variation in the reported level and change in pain over time as shown in the Sankey diagram in Figure 2A. Some participants reported no pain at all throughout, some reported severe pain at all waves, some showed decreasing or increasing levels of pain across time, and, in participants with pain, severity could fluctuate from one wave to the next. Figure 2B shows an illustrative example of 9 participants' pain reports over 11 waves, chosen to demonstrate the diversity of pain trajectories, along with the model estimated trajectories.

The LGCM of pain severity does not provide standard fit indices due to the MLR estimator having to compute latent factors from categorical variables. The standardised estimates demonstrate that, on average, the participants report worsening pain over time. Although the third threshold was free to vary across waves, it remained within a limited range of values (min = 1.029, max = 1.409). Figure 2C shows the model-estimated trajectories of pain severity, with the average trajectory depicted in red and 150 randomly selected individual-level trajectories depicted in grey. This underscores the high variance in the initial pain severity and lower variance in the slope of pain over time.

#### 3.2.2. Cognitive function univariate latent growth curve modelling

The individual LCGM of cognitive tests all demonstrated excellent fit (word recall CFI = 0.956, RMSEA = 0.039; animal naming CFI = 0.976, RMSEA = 0.026; letter cancellation CFI = 0.989, RMSEA = 0.017) and, on average, a decline in test performance over time (word recall intercept β = 3.485, slope β = −0.526; animal naming intercept β = 3.826, slope β = −0.045; letter cancellation intercept β = 4.078, slope β = −0.316; all estimates significant with *P* ≤ 0.008). Model-estimated average and individual-level trajectories for 150 randomly selected participants for each test are shown in supplemental digital content VII, http://links.lww.com/PAIN/C534.

**Figure 3.**
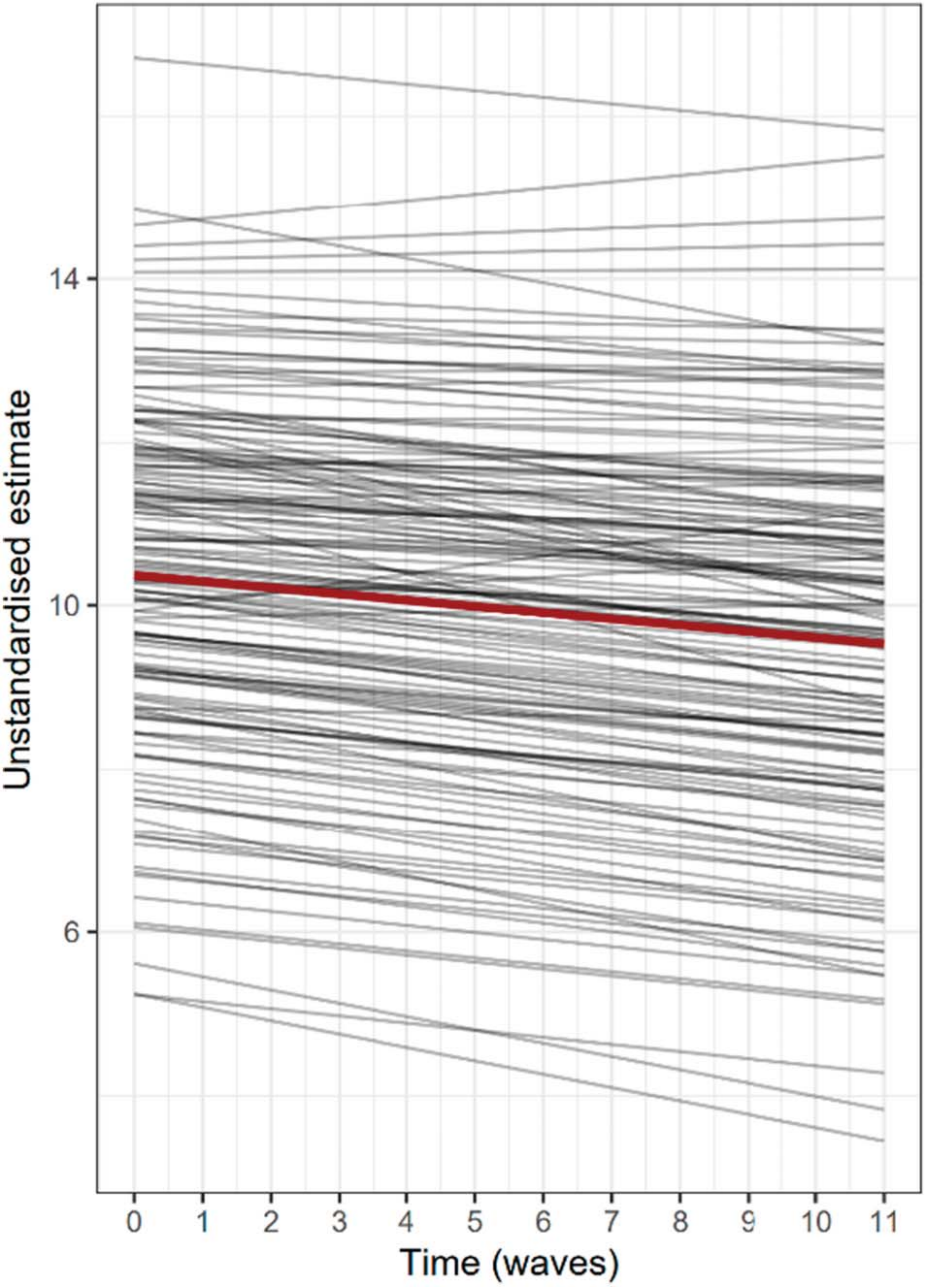
Trajectories of general cognitive function. The estimated trajectory (intercept and slope) of general cognitive function in a random sample of 150 participants estimated by measurement model (shown in grey), and the model's mean estimate shown in dark red (intercept β = 10.364, slope β = −0.076).

The factor-of-curves model of cognitive function showed excellent fit (CFI = 0.973, RMSEA = 0.021) and estimated an average decrease over time in general cognitive performance (standardised estimates for intercept β = 3.978, *P* < 0.001 and slope β = −0.754, *P* < 0.001). Model-estimated average and individual-level trajectories for 150 randomly selected participants are shown in Figure 3. Given that the 3 cognitive tests capture different domains of cognitive function (memory, executive function, and processing speed), variation in the magnitude of the estimated loadings is expected and indicates a different degree of contribution to the higher-order factor. The general cognitive function factors captured nearly all the variance in both word recall (standardised loading on intercept λ = 0.896 and slope λ = 0.853) and animal naming (standardised loading on intercept λ = 0.828 and slope λ = 0.982). In contrast, the letter cancellation's loadings contributed moderately to both higher-order factors (λ = 0.665 on the intercept and λ = 0.633 on the slope, in standardised units), meaning that a greater proportion of its variance is not shared with the other 2 tests. This could also be a result of this variable's high amount of missing data by design or it might reflect on the quality of the test.

Evaluation of rank order stability of pain and cognitive function demonstrated high correlations across waves, indicating that participants' baseline rank tends to be maintained across time (see supplemental digital content VIII, http://links.lww.com/PAIN/C534).

We also conducted sensitivity analyses to investigate the robustness of our findings. When inspecting the means of the latent variables across models, the mean intercept and slope for cognitive function stayed relatively consistent; however, the means of the latent variables of pain severity showed somewhat greater variability. We, therefore, report them alongside each model result.

### 3.3. Longitudinal associations between pain and cognitive function (parallel process latent growth curve modelling)

**Figure 4.**
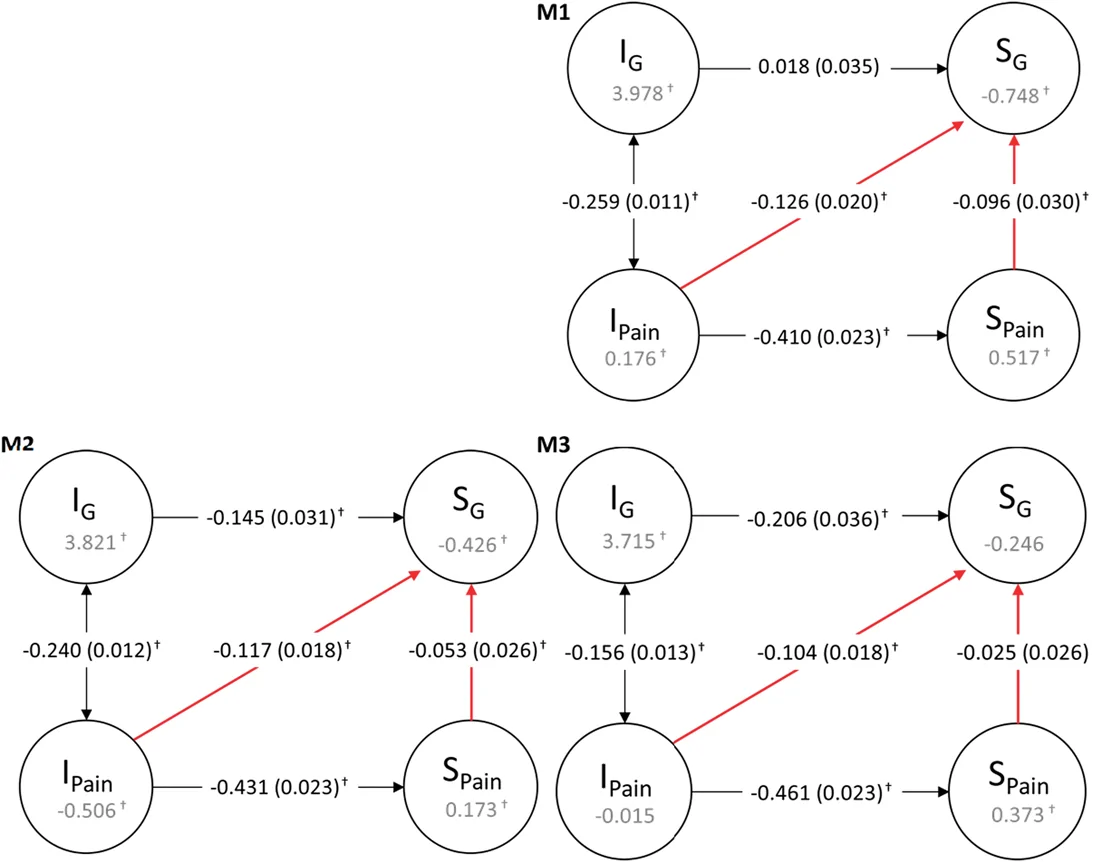
Simplified path diagram of parallel process latent growth curve modelling of pain severity and cognitive function. Model 1 (M1) has not been adjusted for any covariates; model 2 (M2) was adjusted for age and sex; and model 3 (M3) was adjusted for age, sex, ethnicity, socioeconomic status, and comorbidities. I, Intercept, S, Slope, G, General cognitive function. The values inside the circles are the mean estimates. Single-headed arrows are regression paths, and double-headed arrows are correlation paths. All estimates are standardised. Standard errors are in brackets, and † indicates a statistically significant value at α = 0.05 after false discovery rate correction after the Benjamini–Hochberg procedure.

We report below results in standardised units and *P*-values have undergone false discovery rate correction after the Benjamini–Hochberg procedure on each individual model. Summary results for the relationship between pain severity and cognitive function intercept and slope are shown in Figure 4 and Table 3. More detailed results including regression coefficients on the covariates are reported in supplemental digital content IX (http://links.lww.com/PAIN/C534), model fit information are in Supplementary X and longitudinal associations between the latent pain factors and individual test intercepts and slopes are described in supplemental digital content XI (http://links.lww.com/PAIN/C534).

**Table 3:** Summary of results.

Variables	Model 1 (no covariates)			Model 2 (age and sex)			Model 3 (age, sex, ethnicity, socio-economic status, and comorbidities)		
	β	SE	*P*	β	SE	*P*	β	SE	*P*
General cognitive function									
Slope of pain → slope of g	−0.096	0.030	0.001	−0.053	0.026	0.039	−0.025	0.026	0.365
Intercept of pain → slope of g	−0.126	0.020	<0.001	−0.117	0.018	<0.001	−0.104	0.018	<0.001
Intercept of g → slope of g	0.018	0.035	0.604	−0.145	0.031	<0.001	−0.206	0.036	<0.001
Intercept of pain → slope of pain	−0.410	0.023	<0.001	−0.431	0.023	<0.001	−0.461	0.023	<0.001
Intercept of g ↔ intercept of pain	−0.259	0.011	<0.001	−0.240	0.012	<0.001	−0.156	0.013	<0.001
Mean intercept of g	3.978	0.045	<0.001	3.821	0.043	<0.001	3.715	0.062	<0.001
Mean slope of g	−0.748	0.200	<0.001	−0.426	0.134	0.002	−0.246	0.210	0.312
Mean intercept of pain	0.176	0.033	<0.001	−0.506	0.034	<0.001	−0.015	0.069	0.823
Mean slope of pain	0.517	0.027	<0.001	0.173	0.033	<0.001	0.373	0.089	<0.001

Estimates are all standardised.

β, Estimate; SE, standard error; g, general cognitive function as determined by the factor-of-curves method. → represent regression paths, ↔ show correlations. The *P*-values are adjusted within each model for false discovery rate using the Benjamini–Hochberg procedure with α = 0.05.

In the unadjusted model (M1), more initial pain was correlated with lower initial cognitive function (*r* = −0.259, *P* < 0.001) and with accelerated cognitive decline (β = −0.126, *P* < 0.001). More initial pain was strongly associated with worsening pain over time (β = −0.410, *P* < 0.001). Increasing pain severity over time was associated with a faster rate of cognitive decline over time (β = −0.096, *P* = 0.001). Because the model is simultaneously accounting for the effect of initial pain on slopes of pain and *g*, worsening pain over time has an effect on the rate of cognitive decline above and beyond the effect of initial pain. When adjusting for age and sex (M2), pain severity slope remained associated with the slope of cognitive function (β = −0.053, *P* = 0.039), higher levels of initial pain remained correlated with lower initial cognitive function (*r* = −0.240, *P* < 0.001), and remained associated with worsening pain over time (β = −0.431, *P* < 0.001) and accelerated cognitive decline (β = −0.117, *P* < 0.001). When additionally adjusting for ethnicity, socioeconomic status, and comorbidities (M3), the magnitude of the association between pain slope and general cognitive function slope was attenuated to nonsignificance (β = −0.025, *P* = 0.365). In this fully adjusted model, higher initial pain severity remained significantly correlated with lower initial cognitive function (*r* = 0.156, *P* < 0.001) and remained associated with worsening pain over time (β = −0.461, *P* < 0.001) and with steeper cognitive decline over time (β = −0.104, *P* < 0.001).

### 3.4. Sensitivity analyses

To explore our results further, we ran 2 bivariate models where the intercept and slope of pain were not mutually conditioned. We found that the path estimates of these factors on cognitive change remained significant and of similar magnitude (see supplemental digital content XII, http://links.lww.com/PAIN/C534).

To identify which covariates may be driving the attenuation of the slope–slope association in model 3, we fitted nested models adding each set of covariates (ie, comorbidities, socioeconomic status, and ethnicity) individually to the age- and sex-adjusted model (M2): The addition of ethnicity to model 2 did not affect the estimate (β = −0.054, *P* = 0.036); in contrast, both socioeconomic status and comorbidities significantly attenuated the regression of slope of cognitive function on slope of pain (β = −0.035, *P* = 0.172 and β = −0.042, *P* = 0.107 respectively). In all 3 of these nested models, the standard error of the slope–slope coefficient remained constant (SE = 0.026), indicating no loss of power or precision.

Upon reviewer request, we ran a model which included BMI as covariate rather than comorbidities. There was a large amount of missing data on height and weight in the ELSA dataset, which was handled by our model through listwise deletion. For the subsample of participants for which BMI could be computed (n = 15,931), the regression of slope of cognitive function on slope of pain was already attenuated to nonsignificance with just the age and sex adjustment (β = −0.017, *P* = 0.505), indicating that it is not possible to establish the effect of BMI on the slope–slope association using this dataset, and the subset of participants with BMI data likely suffers from selection bias. More details can be found in supplemental digital content XIII (http://links.lww.com/PAIN/C534).

We also tested for nonlinear trajectories for cognitive function and found that a linear model seemed to fit the data well and avoided overfitting. Adding a quadratic term to the LGCMs of individual cognitive tests showed relatively similar fit on the 3 tests (word recall linear: CFI = 0.956, RMSEA 0.039, quadratic: CFI 0.983, RMSEA 0.025—animal naming linear: CFI 0.976, RMSEA 0.026, quadratic: CFI 0.990, RMSEA 0.018—letter cancellation linear: CFI 0.991, RMSEA 0.020, quadratic: CFI 0.991, RMSEA 0.019); however, MPlus was unable to estimate standard errors when combined into a quadratic factor-of-curves.

To assess the robustness of the results, we ran 2 more sets of parallel process LGCM models: the first was run without partial invariance of the thresholds for the pain severity categorical variable; for the second set of models, we used only waves 1 to 9 to examine the effect of pain severity on cognitive function before the COVID-19 pandemic (the time elapsed between waves 9, 10, and 11 became more irregular). Both sets of models showed very similar regression and correlation path coefficients to the primary analysis (see supplemental digital content XIII, http://links.lww.com/PAIN/C534).

## 4. Discussion

In this study of nearly 20,000 middle-aged and older adults residing in England, followed for more than 20 years, we examined how longitudinal variations in pain severity relate to changes in cognitive function. We found evidence that worsening pain over time was associated with steeper cognitive decline, independently of baseline pain level, and partially attenuated by age and sex. This association was further attenuated and nonsignificant when additionally adjusting for socioeconomic status (SES), ethnicity, and comorbidities although downstream analyses indicate that ethnicity did not contribute to the attenuation. Our models also indicate that initial levels of pain severity were consistently associated with faster rates of decline in cognitive function, even when adjusting for sociodemographic and health variables. We conclude that both the intensity of initial pain and its worsening over time relate to a steeper decline in cognition independently of each other; however, the association between increasing pain severity and cognitive decline is partly explained by the cumulative effects of pain at baseline, sex, age, SES, and comorbid chronic conditions.

The present work is one of only a few studies to have examined change in both chronic pain and cognitive function over time. Our findings substantiate the conclusions drawn by He et al.^20^ Using group-based trajectory modelling and linear mixed effects, they found that the high-stable and moderate-increasing categories of probability of pain were associated with steeper cognitive decline over subsequent 8 to 12 years. We expand these results by capturing variations in pain severity rather than pain presence and by using LGCM to directly link within-person change in pain to within-person change in cognitive function over a 22-year time period. Within-person changes were also captured by Milani et al.^33^ in their cross-lagged analysis of older Puerto Rican adults. They too found greater cognitive decline in participants who had pain at follow-up compared with those who did not; however, this association was found using only 2 waves of data, and over one-third of their sample participants were excluded from the analysis due to missing data. Our study was able to retain its large sample size by leveraging SEM's Full Information Maximum Likelihood (FIML) method. To the best of our knowledge, ours is the first study to examine the association of pain and cognitive function using LGCM and FIML, thereby maintaining representativeness and reducing selection bias.

Another key strength ensuing from our use of LGCM is the factor-of-curves, which has several methodological advantages over previous studies. Techniques that average standardised scores to create a global measure of cognitive function implicitly treat each test as equally reliable, fail to account for measurement error, and discount the shared structure of cognitive function. In contrast, the factor-of-curves method allows us to simultaneously estimate latent intercepts and slopes from multiple cognitive tests and aggregate these into a latent higher-order cognitive factor. This approach not only explicitly accounts for measurement error in repeated indicators but also accurately captures the well-replicated hierarchical structure of cognitive changes over time.^2,18,46,58^ Our cognitive battery included measures of long-term memory, executive function, and processing speed; domains that are sensitive to age-related cognitive decline^56^ and have also been shown to be lower in individuals with chronic pain.^7,8^ Although other important cognitive domains were not assessed, such as visuospatial ability or reasoning, a general measure of cognitive function derived from a small but varied set of tests, such as we have here, has previously been found to provide a broadly comparable summary across different combinations of measures.^23,24^ Thus, our method provides a more robust, theoretically sound, and psychometrically valid estimation of cognitive change than was previously achieved.

As one of the largest studies with one of the longest follow-ups in the literature, our work also consolidates the evidence on the predictive nature of cross-sectional measures of pain on cognitive decline.^6,22,25,47,49,63^ In many of these studies, pain is defined as chronic (lasting at least 3 months) or persistent by design, where 2 pain measurements define the variable. A limitation of the present study's pain severity measure is that we cannot directly confirm, at each wave, whether the pain experienced is chronic. Indeed, “Are you often troubled by pain?” does not specify a time frame or require participants to answer if they are currently experiencing pain. In their work on disability, Banks et al.^4^ found that although 60% of adult Dutch respondents reported pain in the last 30 days, only a quarter of the sample answered yes to “are you often troubled with pain.” This phrasing might, therefore, selectively omit transient or minor episodes and more reliably capture persistent pain. Furthermore, the repetition of assessments up to 11 times over 22 years is a key strength of this study. Studies such as Van der Leeuw et al.'s^30^ investigated pain over a shorter period of time and found no association with cognitive decline; therefore, our measure of baseline pain severity is likely capturing a chronic characteristic of the condition. In contrast, variation in within-person changes in pain over time were modest, as is typically observed in longitudinal studies, and small relative to baseline variance. Consequently, any effects on cognitive decline produced by longitudinal change in pain, if present, could be subtle and difficult to detect.

Beyond ELSA's large sample size, ensuring sufficient statistical power to find associations even in complex modelling, the cohort is also nationally representative of adults aged over 50 years living in England. The study continued follow up with participants who moved from the community to care homes; hence, our results are generalisable to frailer groups of older adults. ELSA's use of a 4-point scale to measure pain severity might also be a limitation. This measure may be less sensitive to subtle but important fluctuations in pain over time than the typical 11-point (0-10) numerical rating scale.^9,37,40^ The low counts in the severe pain category within ELSA led the estimator to struggle to translate the observed variable to latent factors and compelled us to allow partial invariance of the thresholds. This likely drove the variability we observed in mean estimates of the pain intercept and slope across the several models run in sensitivity analysis. Nonetheless, estimated correlation and regression paths from pain intercept and slope to cognitive function intercept and slope were remarkably consistent across all parallel process models conducted, further affirming the conclusions drawn in the present study.

As one of the first studies to fully explore the longitudinal relationship between pain severity and cognitive decline, we selected covariates parsimoniously. The SES variable was based on individual occupation. Although this marker may be a less accurate index of household income, our focus rests on individual occupation, which is more directly associated with pain^39^ and cognitive function.^45,59^ We also found ELSA's design somewhat restrictive. For example, height and weight were scarcely measured across waves, so to avoid selection bias, we decided to adjust for comorbidities, which were likely to highly correlate with BMI such as diabetes, hypertension, and heart disease. Still, limited availability of data on these covariates across waves and substantial missingness meant that their temporal changes could not be accounted for in the current study. As per our sensitivity analyses, comorbidities seemed to weaken the association of worsening pain severity with steeper cognitive decline to nonsignificance. Socioeconomic status also had a considerable attenuating effect on this association. Mediation analyses were beyond the scope of this study; however, applied to these variables, they may point to a potential mechanism underlying the conclusions of our analyses. Future research should investigate potential pathways through which pain is associated with cognitive decline, including other measures such as sleep disturbances, psychiatric conditions, and prescription medications.

This study focused on only one measure of worsening pain over time, namely, increasing pain severity. Other indicators of worsening pain, such as duration of pain, the spread of pain to additional body sites, or increasing pain interference, may demonstrate a different pattern of association with cognitive decline. Pain interference (ie, whether pain interferes with the ability to perform work or household chores) is a strong predictor of cognitive impairment^5,26^ but was not assessed in the ELSA cohort. A possible reverse effect where people with declining cognitive function might report more pain or struggle more to cope with symptoms than those without cognitive impairment also requires further scrutiny. Investigating these other measures of pain over time and their association with cognitive function (in either direction) would be useful next steps to follow from our work.

## 5. Conclusion

Persistent pain can be debilitating and is associated with many negative health outcomes. In a sample of nearly 20,000 middle-aged and older adults, followed up for over 20 years, we found that increasing pain over time was associated with steeper rates of cognitive decline and partly attenuated by sociodemographic variables and concurrent health conditions. Our study shows that higher initial pain is associated with accelerated cognitive decline, further elucidating the cognitive consequences of chronic pain and highlighting the importance of preventing its onset.

## Conflict of interest statement

The authors have no conflict of interest to declare.

## Supplementary Material

## Appendix A. Supplemental digital content

Supplemental digital content associated with this article can be found online at http://links.lww.com/PAIN/C534.
